# Treatment outcomes of antegrade versus retrograde approaches in tandem occlusion: a single-center retrospective study from Vietnam

**DOI:** 10.3389/fneur.2026.1756539

**Published:** 2026-06-24

**Authors:** Tuan Anh Tran, Ton Duy Mai, Anh Minh Nguyen, Hoa Thi Truong

**Affiliations:** 1 Department of Stroke and Cerebrovascular Disease, VNU University of Medicine and Pharmacy, Vietnam National University, Hanoi, Vietnam; 2 Radiology Center, Bach Mai Hospital, Hanoi, Vietnam; 3 Stroke Center, Bach Mai Hospital, Hanoi, Vietnam

**Keywords:** tandem occlusion, stroke, antegrade approach, retrograde approach, stent, balloon angioplasty

## Abstract

**Purpose:**

Tandem occlusion, characterized by concurrent extracranial and intracranial arterial occlusion, is associated with unfavorable clinical outcomes. Despite evidence from observational studies, multicenter registries, and meta-analyses comparing antegrade and retrograde endovascular approaches, the optimal treatment sequence remains unresolved. This study aimed to compare the clinical and angiographic outcomes of antegrade and retrograde strategies in patients with tandem occlusion in Vietnam.

**Method:**

A retrospective single-center study included 74 patients with tandem occlusion treated between January 2023 and June 2025 at Bach Mai Hospital, Hanoi. Patients were grouped into antegrade and retrograde treatment strategies. Demographic, clinical, imaging, and procedural characteristics were collected. The primary outcome was favorable functional outcome at 90 days [modified Rankin Scale (mRS) ≤ 2]. Secondary outcomes included successful reperfusion (mTICI ≥2b), early neurological improvement (NIHSS reduction ≥8 points), symptomatic intracerebral hemorrhage (sICH), and mortality.

**Result:**

The median age was 67 years (IQR 60.75–75), and 81.1% were male. Hypertension was present in 27/74 patients (36.5%). The predominant intracranial occlusion site was the M1 segment (63/74, 85.1%). The median groin puncture–to–reperfusion time was 53 min (IQR 39.75–85.25). Baseline NIHSS scores were similar between the two groups, and ASPECTS ≥6 was observed in 66 of 74 patients (89.2%). Successful reperfusion was achieved in 94.6% of the antegrade group and 91.9% of the retrograde group. Early stent occlusion occurred in 25.7% of patients, and delayed stent occlusion occurred in 4.1% of patients. Favorable functional outcome at 90 days occurred in 45.9% versus 40.5% (*p* = 0.639). sICH occurred in 8.1% of patients (*p* = 0.999), and mortality was 10.8% (*p* = 0.999).

**Conclusion:**

No significant differences were observed between the two approaches in achieving successful reperfusion. Overall, the antegrade and retrograde strategies demonstrated comparable outcomes in reperfusion success, early neurological improvement, and 90-day functional recovery, with no clear clinical advantage for either technique. Accordingly, procedural selection may depend on operator preference and patient-specific anatomical factors. However, given the retrospective single-center design and limited sample size, further multicenter randomized controlled trials are needed to determine the optimal procedural sequence for tandem occlusions.

## Introduction

Tandem lesions (TLs) occur in approximately 20%–30% of patients with acute ischemic stroke caused by large vessel occlusion and are characterized by concurrent extracranial and intracranial arterial occlusion [Di Donna et al. (1)]. These lesions typically involve severe stenosis or occlusion of the cervical internal carotid artery, most commonly due to atherosclerotic disease or arterial dissection, together with a thromboembolic occlusion of the intracranial terminal internal carotid artery or one of its major branches, particularly the middle cerebral artery (2). Patients with tandem lesions generally experience poorer clinical outcomes and prognosis compared with those who present with isolated intracranial occlusion (3).

Despite the clinical significance of TLs, the optimal endovascular treatment strategy remains controversial, and no clear recommendations are specified in current stroke guidelines. A multicenter retrospective study reported that the retrograde approach achieved a significantly higher rate of successful reperfusion than the antegrade approach (92% versus 56%, *p* < 0.001). In the same study, the proportion of patients achieving a favorable 90-day functional outcome was also higher in the retrograde group (44% versus 30%, *p* < 0.05) (4). Similarly, a systematic review and meta-analysis of 11 studies revealed that the retrograde strategy was associated with better angiographic and functional outcomes, including higher successful reperfusion rates (83.8% versus 78.0%, *p* = 0.04) and improved functional independence (47.3% versus 40.2%, *p* = 0.002), with no difference in safety profiles compared with the antegrade technique (5). In contrast, data from a prospectively collected thrombectomy registry across 11 institutions found no significant differences between the two approaches in terms of complete reperfusion, symptomatic intracerebral hemorrhage, 90-day independence, or mortality (6). In Vietnam, a case series of 17 patients published 5 years ago showed that the antegrade approach was associated with lower rates of successful reperfusion and 90-day independence, as well as a higher rate of procedural complications compared with the retrograde strategy (7).

Importantly, this is not a new research question. Several observational studies, including large multicenter analyses from the TITAN investigators and other international groups, have compared intracranial-first (“head-first” or retrograde) and extracranial-first (“neck-first” or antegrade) strategies in tandem occlusions. However, the available evidence remains insufficient to establish the superiority of either approach, and overall has not shown a clear or consistent advantage of one procedural strategy over the other (6). Earlier meta-analyses, including the study by Wilson et al., likewise reported no statistically significant differences between extracranial-first and intracranial-first approaches (8).

Data from low and middle-income countries, including Vietnam, remain scarce, and no prior study has systematically compared antegrade and retrograde strategies in this setting. Accordingly, our study was designed as a single-center retrospective analysis intended to add real-world data from Vietnam rather than to definitively resolve this question. Given the inconsistencies in the existing literature and the absence of guideline recommendations, we conducted a study in a Vietnamese cohort with tandem large vessel occlusion to compare the clinical and angiographic outcomes of antegrade and retrograde endovascular approaches.

## Materials and methods

### Study design

This retrospective study was conducted at Bach Mai Hospital, Hanoi, Vietnam, from January 2023 to June 2025. Patients were eligible for endovascular thrombectomy if they met the following inclusion criteria: (1) age 18 years or older; (2) indication for thrombectomy according to national stroke guidelines; (3) confirmation of tandem occlusion by digital subtraction angiography, defined as concurrent extracranial internal carotid artery (ICA) lesion and intracranial occlusion of the middle cerebral artery; (4) documented symptoms with available clinical outcome at 90 days; and (5) availability of high quality imaging, including MRI performed within 24 h post-intervention, and complete medical records with follow up information at 3 months. Exclusion criteria included: (1) failure to meet the inclusion criteria; (2) refusal by the patient’s family; (3) insufficient clinical or imaging data; and (4) absence of post-intervention MRI.

### Data collection

Baseline variables included age, sex, vascular risk factors, and National Institutes of Health Stroke Scale (NIHSS) score at admission, Alberta Stroke Program Early CT Score (ASPECTS), occlusion site, thrombectomy technique, and procedural duration. Decisions regarding the endovascular strategy (antegrade versus retrograde), the use of carotid stenting, and pre or post-stenting balloon angioplasty were made by the neurointerventionalists. Intravenous thrombolysis was administered when indicated using recombinant tissue plasminogen activator (IV r-tPA) in accordance with national guidelines and neurologist assessment, including from onset time to hospital admission time less than 4.5 h, no contraindication with IV r-tPA.

### Endovascular treatment

**Figure 1:**
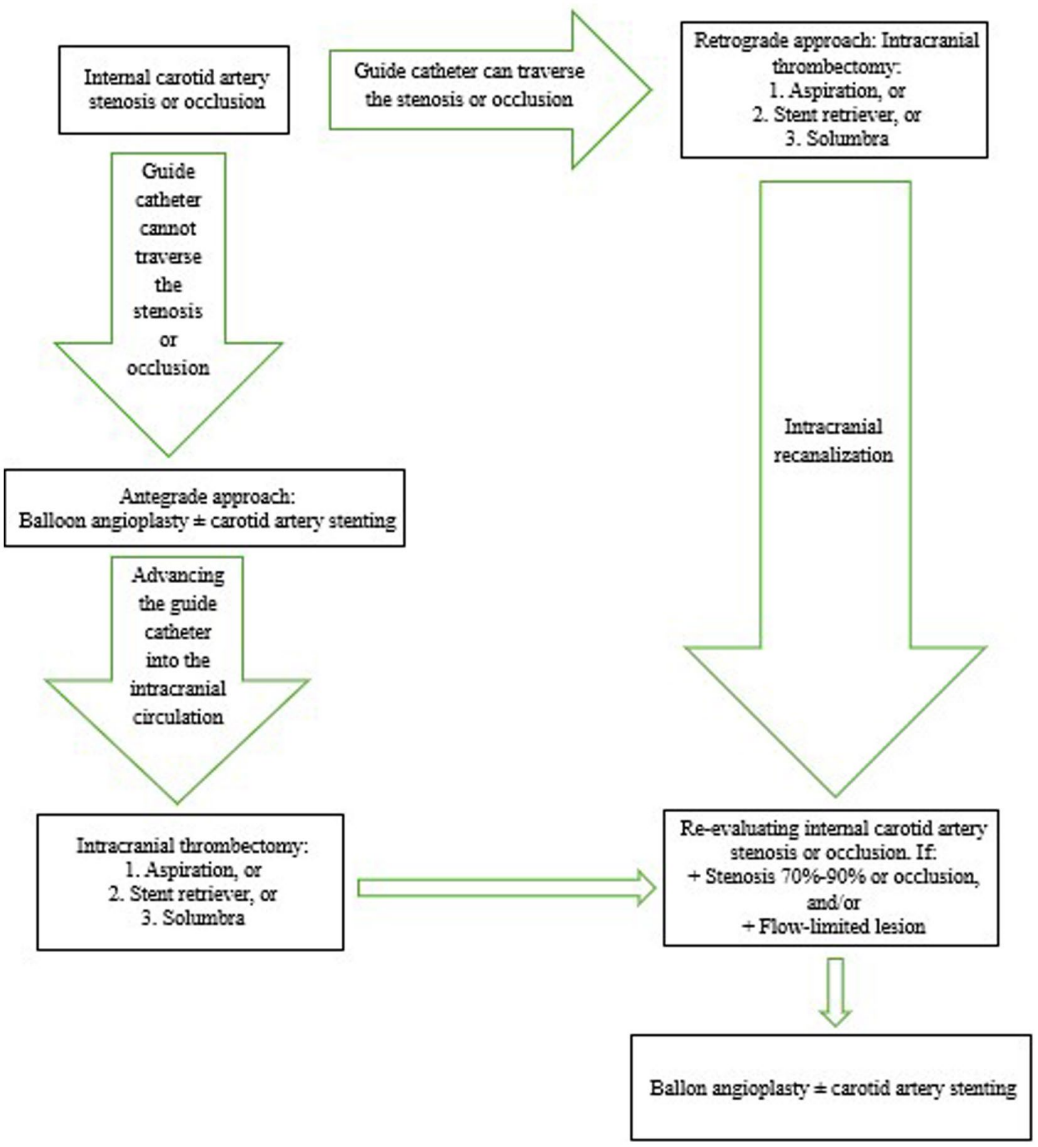
Flowchart of endovascular treatment strategies for tandem occlusion stroke. The choice between the antegrade approach (extracranial-first) and the retrograde approach (intracranial-first) is determined by the ability of the guide catheter to traverse the proximal cervical internal carotid artery stenosis or occlusion.

Endovascular treatment was performed by neurointerventionalists with at least 5 years of experience in neurointervention and/or who had performed at least 1,000 stroke cases. Interventional strategies were determined based on anatomical factors and technical feasibility, including the degree of cervical carotid artery stenosis, the ability of guide catheter to traverse the lesion, and the accessibility of the intracranial lesion to the equipment. The flowchart in Figure 1 illustrates the key technical steps in the two groups.

### General procedure

Intravenous thrombolysis (IV r-tPA) was administered prior to endovascular thrombectomy (EVT) when not contraindicated. Contraindications included a history of ischemic stroke within the previous 3 months; severe head trauma within 3 months; intracranial or spinal surgery within 3 months; platelet count <100,000/mm^3^; INR > 1.7, aPTT >40 s, or PT > 15 s; therapeutic-dose low-molecular-weight heparin within the preceding 24 h; recent use of a direct thrombin inhibitor or factor Xa inhibitor when coagulation parameters had not normalized or when the required drug-free interval of at least 48 h had not been met; infective endocarditis; aortic arch dissection; and intra-axial intracranial neoplasm (9). General anesthesia was reserved for clinically unstable patients, including those with decreased level of consciousness (GCS < 9), airway compromise, severe agitation, or uncontrolled vomiting. All procedures were performed using a biplane digital subtraction angiography system (Philips Azurion 7 B20) by neurointerventionalists with postgraduate training at Bach Mai Hospital—a tertiary referral general hospital and a major referral center in northern Vietnam. All operators had more than 5 years of experience in neurovascular intervention and were licensed to perform neurointerventional procedures in accordance with regulations of the Vietnamese Ministry of Health. The choice between an antegrade and a retrograde strategy was left to the discretion of the treating operator, based on lesion anatomy and technical feasibility, including the degree of cervical internal carotid artery stenosis or occlusion, the ability to cross the lesion, and the feasibility of accessing the intracranial occlusion.

Unfractionated heparin was administered intra-procedurally when carotid stenting was performed, typically at a dose of 50–70 IU/kg. Patients undergoing carotid artery stenting received peri-procedural antiplatelet loading with aspirin 300 mg and clopidogrel 150 mg. Carotid stenting was performed using the Protégé™ RX Self-Expanding Carotid Stent System (Medtronic), an open-cell self-expanding stent. When incomplete stent expansion was observed, adjunctive balloon angioplasty was performed at the operator’s discretion to optimize stent apposition and luminal expansion. Tirofiban was not used because it was unavailable at our center.

Follow-up non-contrast brain CT was routinely performed 16–24 h after the procedure. In the absence of hemorrhagic transformation, dual antiplatelet therapy was continued with aspirin 75–100 mg daily plus clopidogrel 75 mg daily. In patients with hemorrhagic infarction type 1 or 2 (HI1–2), antiplatelet therapy was de-escalated to aspirin 81 mg daily with close clinical and imaging surveillance. In patients with parenchymal hematoma type 1 or 2 (PH1–2) or symptomatic hemorrhagic transformation, dual antiplatelet therapy and anticoagulation were withheld, acknowledging the increased risk of in-stent thrombosis. After hospital discharge, patients were scheduled for follow-up within 1–3 months, with carotid duplex ultrasonography and computed tomography angiography performed to assess stent patency and carotid artery status.

### Antegrade approach

A large-bore guide catheter (Neuron MAX 088, Penumbra; Fubuki 8F, Asahi) was positioned in the common carotid artery. A 0.035-inch guidewire supported by a JB2 catheter was initially used to traverse the cervical internal carotid artery (ICA) stenosis or occlusion; if unsuccessful, a 0.014-inch microwire (Chikai, Asahi) was used. Contrast injection was performed to confirm true lumen access. In the antegrade group, carotid artery stenting was performed more frequently, as most patients presented with severe cervical ICA stenosis. Stent placement was indicated for cervical ICA stenosis >70%, or for stenosis <70% in the presence of persistent slow flow or inadequate perfusion after a 10-min observation period. In such cases, the extracranial lesion was treated first with balloon angioplasty and stent deployment before intracranial thrombectomy. Balloon angioplasty was selectively performed using 3.0–4.0 mm balloons (EverCross, Medtronic; Viatrac, and Abbott), primarily in patients with carotid occlusion requiring restoration of luminal patency before stent placement, or in those with <70% cervical ICA stenosis in whom predilation was necessary to facilitate device passage for subsequent carotid and intracranial intervention. Intracranial recanalization was then achieved using direct aspiration and/or stent retriever thrombectomy (Solitaire X, Trevo XP, Eric, and Tigertriever). When a stent retriever was used, it was withdrawn into the aspiration catheter before reaching the guide catheter to minimize contact with the carotid stent. Aspirin 300 mg was administered as peri-procedural antiplatelet loading following intra-procedural heparin administration. When an acute intra-procedural intracranial arterial hemorrhage or perforation occurred while systemic heparinization was active, our institutional rescue protocol required immediate intervention through the following sequential steps: first, administration of protamine sulfate (at a dose of 1 mg per 100 units of unfractionated heparin administered) is performed to rapidly reverse the systemic effect of heparinization. Next, immediate inflation of a compliant balloon at the site of injury is maintained for 5–10 min to achieve mechanical hemostasis. Finally, if bleeding persists despite repeated rounds of balloon inflation, sacrifice of the injured segment with detachable coils or liquid embolic agents (glue) is considered as a final rescue measure.

### Retrograde approach

The proximal lesion was initially crossed using a microwire, followed by advancement of an aspiration catheter (Sofia Plus, Esperance, and React 68). The catheter was positioned adjacent to the thrombus, and mechanical thrombectomy was performed using the same technique as described above. After successful intracranial recanalization, a 300-cm microwire (Chikai, Asahi) was introduced to facilitate carotid stenting when indicated. Stenting and balloon angioplasty were then performed according to the same indications and technique as in the antegrade approach. If the aspiration catheter could not be advanced across the proximal cervical ICA lesion despite multiple attempts, the procedure was converted to an antegrade strategy, with proximal angioplasty and/or stenting performed before intracranial thrombectomy. Final angiography was used to confirm carotid stent placement and intracranial reperfusion. Aspirin 300 mg was administered as peri-procedural antiplatelet loading following intra-procedural heparin administration.

### Outcome measures

#### Angiographic and clinical outcomes

Angiographic results were graded using the modified Thrombolysis in Cerebral Infarction (mTICI) scale, with successful reperfusion defined as mTICI 2b–3. Symptomatic intracranial hemorrhage (sICH) was defined as any intracranial extravascular blood associated with neurological deterioration, indicated by an increase of eight or more points on the NIHSS within 24 h, according to the Second European Cooperative Acute Stroke Study II criteria.

Early stent occlusion was defined as the complete occlusion of the implanted carotid stent occurring within 24 h after the procedure, as confirmed by follow-up vascular imaging. Late stent occlusion was defined as the complete occlusion of the implanted carotid stent occurring more than 24 h after the procedure.

Functional outcomes were evaluated using the modified Rankin Scale (mRS) at discharge and at 90 days by a certified stroke neurologist. Functional independence was defined as an mRS score of 0–2 at 90 days.

#### Postprocedural imaging

All patients underwent CT or MRI within 18 ± 6 h after the procedure to assess hemorrhagic transformation or intracranial hemorrhage following European Cooperative Acute Stroke Study II criteria.

#### Early neurological improvement

Early neurological improvement was defined as a reduction of ≥8 points on the NIHSS compared with baseline or an NIHSS score of 0–1 at 24 h post procedure.

### Statistical analysis

Descriptive variables were reported as mean ± standard deviation for normally distributed data or median with interquartile ranges for non-standard distributed data. In the univariate analysis, differences between treatment groups were assessed using the Mann–Whitney U test, Fisher’s exact test, or the chi-square test as appropriate. Statistical analyses were performed using SPSS version 27.0. A *p*-value <0.05 was considered statistically significant, and 95% confidence intervals (CI) were calculated where applicable.

### Ethical approval

The study protocol was approved by the Institutional Ethics Committee of Bach Mai Hospital (Approval No. 83/BM-HDDD).

## Results

### Demographic and clinical information

A total of 74 patients met the inclusion criteria between January 2023 and June 2025. The median age was 67 years (IQR 60.75 to 75), and 81.1% (60/74) were male. Hypertension was present in 36.5% of patients. Other comorbidities, including diabetes mellitus, dyslipidemia, atrial fibrillation, heart failure, chronic kidney disease, and previous myocardial infarction, were each observed in fewer than 10% of the cohort (Table 1).

**Table 1:** Baseline characteristics of patients treated with antegrade versus retrograde approaches.

Variable	Overall (*n* = 74)	Treatment strategy			
		Antegrade (*n* = 37)	Retrograde (*n* = 37)	95% CI	*p*-value
Demographic characteristics					
Age, years, median (IQR)	67 (60.75–75)	66 (60–75.5)	70 (61–72.5)	NA	0.75^a^
Gender, male, *n* (%)	60 (81.1)	31 (83.8)	29 (78.4)	0.441–4.608	0.553^b^
Past medical history					
Diabetes mellitus, *n* (%)	6 (8.1)	5 (13.5)	1 (2.7)	0.624–50.725	0.088 ^c^
Hypertension, *n* (%)	27 (36.5)	11 (29.7)	16 (43.2)	0.213–1.449	0.227 ^b^
Dyslipidemia, *n* (%)	4 (5.4)	2 (5.4)	2 (5.4)	0.133–7.502	0.999 ^c^
Atrial fibrillation, *n* (%)	2 (2.7)	0 (0)	2 (5.4)	1.622–2.609	0.493 ^c^
Heart failure, *n* (%)	2 (2.7)	2 (2.7)	2 (2.7)	0.06–16.611	0.999 ^c^
Chronic kidney disease, *n* (%)	2 (2.7)	2 (5.4)	0 (0)	1.622–2.609	0.493 ^c^
Old myocardial infarction, *n* (%)	2 (2.7)	2 (2.7)	2 (2.7)	0.06–16.611	0.999 ^c^
Clinical characteristics					
Hemiplegia, *n* (%)	72 (97.3)	36 (97.3)	36 (97.3)	0.06–16.611	0.999 ^c^
Facial nerve palsy, *n* (%)	3 (4.1)	2 (5.4)	1 (2.7)	0.178–23.723	0.999 ^c^
Aphasia, *n* (%)	4 (5.4)	3 (8.1)	1 (2.7)	0.315–32.039	0.615^c^
Confusion, *n* (%)	1 (1.4)	0 (0)	1 (2.7)	1.607–2.559	0.999^c^
Altered mental status, *n* (%)	2 (2.7)	1 (2.7)	1 (2.7)	0.06–16.611	0.999^c^
NIHSS at admission, median (IQR)	13 (10–16)	13 (8.5–15)	13 (10–16)	NA	0.983^a^
GCS score at admission ≥13, *n* (%)	8 (10.8)	4 (10.8)	4 (10.8)	0.231–4.338	0.999^c^
Imaging characteristics					
Site of occlusion, left, *n* (%)	37 (50)	19 (51.4)	18 (48.6)	0.448–2.773	0.816^b^
Occlusion of M1, *n* (%)	63 (85.1)	34 (91.9)	29 (78.4)	0.785–12.888	0.102^b^
Occlusion of M2, *n* (%)	8 (10.8)	3 (8.1)	5 (13.5)	0.125–2.558	0.711^c^
Occlusion of M1 and M2, *n* (%)	3 (4.1)	0 (0)	3 (8.1)	1.638–2.662	0.24^c^
Atherosclerotic obstruction, *n* (%)	56 (75.7)	27 (73)	29 (78.4)	0.256–2.166	0.588^b^
Arterial dissection obstruction, *n* (%)	14 (18.9)	10 (27)	4 (10.8)	0.861–10.839	0.075^b^
Thrombotic obstruction, *n* (%)	4 (5.4)	0 (0)	4 (10.8)	1.655–2.718	0.115^c^
ASPECTS ≥6, *n* (%)	66 (89.2)	34 (91.9)	32 (86.5)	0.391–8.021	0.711^c^
Collateral score ≥2, *n* (%)	51 (68.9)	25 (67.6)	26 (70.3)	0.329–2.361	0.802^b^
Treatment details and process times					
IVT, *n* (%)	16 (21.6)	8 (21.6)	8 (21.6)	0.331–3.025	0.999^b^
Use of balloon angioplasty, *n* (%)	60 (81.1)	37 (100)	23 (62.2)	0.278–0.528	**<0.001** ^**c**^
Use of stent in internal carotid artery, *n* (%)	46 (62.2)	34 (91.9)	12 (32.4)	6.021–92.591	**<0.001** ^**b**^
Onset to admission, hours, median (IQR)	3.865 (2.207–6.032)	3.5 (2.195–6.165)	4 (2.27–6.065)	NA	0.791 ^a^
Onset to puncture, hours, median (IQR)	5.94 (4.375–9.377)	5.67 (4.325–8.8)	6.25 (4.215–9.675)	NA	0.851 ^a^
Admission to MRI/MSCT time, minutes, median (IQR)	30.5 (19.75–41.75)	29 (20.5–40.5)	32 (19–48)	NA	0.816 ^a^
Admission to groin puncture time, hours, median (IQR)	2.245 (1.725–2.862)	2.25 (1.565–3.11)	2.2 (1.75–2.775)	NA	0.799 ^a^
Groin puncture to reperfusion, minutes, median (IQR)	53 (39.755–85.25)	54 (36.5–85.5)	50 (42.5–85.5)	NA	0.758 ^a^

a: U Mann–Whitney.

b: *χ*^2^ test.

c: Fisher’s exact test.

NA, not applicable; NIHSS, National institutes of health stroke scale; ASPECTS, Alberta stroke program early CT score; IVT, Intravenous thrombolysis; GCS, Glasgow coma scale; MRI, Magnetic resonance imaging; MSCT, Multislice computed tomography.

Hemiplegia was the predominant presenting symptom, occurring in 97.3% of patients (72/74). Other neurological signs, such as facial nerve palsy, aphasia, confusion, and altered mental status, were uncommon, ranging from 1.4 to 5.4%. Intravenous thrombolysis was administered in 21.6% of cases (16/74).

Regarding imaging characteristics, 85.1% of patients (63/74) had an occlusion of the M1 segment of the middle cerebral artery. Baseline stroke severity and imaging metrics were comparable between the antegrade and retrograde groups. There were no significant differences between the two groups in baseline NIHSS, Glasgow Coma Scale score ≥13, ASPECTS ≥6, or collateral score ≥2. The median NIHSS score at admission was 13 (IQR 10–16), and ASPECTS ≥6 was observed in 89.2% of patients.

Process time metrics showed that the median admission-to-MRI or MSCT time was 30.5 min (IQR 19.75 to 41.75). The median groin puncture-to-reperfusion time was 53 min (IQR 39.75 to 85.25). This interval was similar between the two treatment strategies: 54 min (IQR 36.5 to 85.5) in the antegrade group and 50 min (IQR 42.5 to 85.5) in the retrograde group.

### Procedural outcome

Table 2 presents the procedural outcomes for both treatment strategies. Early stent occlusion occurred in 19 of 74 patients (25.7%), with similar rates between the two groups (25.7% in the antegrade group and 29.7% in the retrograde group). Successful reperfusion, defined as a modified Thrombolysis in Cerebral Infarction score of 2b to 3, was achieved in 69 of 74 patients (93.2%). The rates were comparable between groups, with 94.6% in the antegrade group and 91.9% in the retrograde group.

**Table 2:** Procedural outcomes following intervention in the two treatment strategies.

Variable	Overall (*n* = 74)	Treatment strategy			
		Antegrade (*n* = 37)	Retrograde (*n* = 37)	95% CI	*p*-value
Successful reperfusion, mTICI ≥2b, *n* (%)	69 (93.2)	35 (94.6)	34 (91.9)	0.243–9.824	0.999^c^
Improvement in NIHSS after 24 h, NIHSS reduction of 8 points, *n* (%)	34 (45.9)	16 (43.2)	18 (48.6)	0.448–2.773	0.641^b^
Symptomatic intracerebral hemorrhage (ECASS II), *n* (%)	6 (8.1)	3 (8.1)	3 (8.1)	0.188–5.309	0.999^c^
Post-procedural stenosis < 30%, *n* (%)	37 (50)	19 (51.4)	18 (48.6)	0.448–2.773	0.999^c^
Early stent occlusion, *n* (%)	19 (25.7)	8 (21.6)	11 (29.7)	0.227–1.87	0.425^b^

b: *χ*^2^ test.

c: Fisher’s exact test.

mTICI, modified Thrombolysis in Cerebral Infarction; NIHSS, National Institutes of Health Stroke Scale; ECASS II, European Cooperative Acute Stroke Study II.

Early neurological improvement, defined as a reduction of at least 8 points in the National Institutes of Health Stroke Scale score at 24 h, was observed in 34 patients (45.9%). The proportion was 43.2% in the antegrade group and 48.6% in the retrograde group.

Symptomatic intracerebral hemorrhage occurred in 6 patients (8.1%). The incidence was identical in both groups, each with 3 cases (8.1%).

Postprocedural stenosis of less than 30% was documented in 37 patients (50.0%). This occurred in 19 patients (51.4%) in the antegrade group and 18 patients (48.6%) in the retrograde group.

### Clinical outcomes at 90 days

Table 3 presents the 90-day clinical outcomes for the two treatment strategies. There were no statistically significant differences between the antegrade and retrograde groups in functional outcomes or mortality.

**Table 3:** Clinical outcomes at 90 days in the two treatment strategies.

Variable	Overall (*n* = 74)	Treatment strategy			
		Antegrade (*n* = 37)	Retrograde (*n* = 37)	95% CI	*p*-value
Late stent occlusion, *n* (%)	3 (4.1)	1 (2.7)	2 (5.4)	0.046–5.606	0.999^c^
Favorable clinical outcome 90 days, mRS ≤ 2, *n* (%)	32 (43.2)	17 (45.9)	15 (40.5)	0.496–3.132	0.639^b^
Mortality, *n* (%)	8 (10.8)	4 (10.8)	4 (10.8)	0.231–4.338	0.999^c^

b: *χ*^2^ test.

c: Fisher’s exact test.

mRS, modified Rankin Scale.

A favorable clinical outcome, defined as a modified Rankin Scale score of 0 to 2 at 90 days, was achieved in 32 of 74 patients (43.2%). The proportion was 45.9% in the antegrade group and 40.5% in the retrograde group (*p* = 0.639). Delayed stent occlusion was rare, occurring in 3 patients (4.1%) overall, with comparable rates in the antegrade and retrograde groups (2.7% vs. 5.4%).

The overall mortality rate at 90 days was 10.8%, and the rate was identical in both groups, with 4 patients (10.8%) in the antegrade group and 4 patients (10.8%) in the retrograde group (*p* = 0.999).

**Figure 2:**
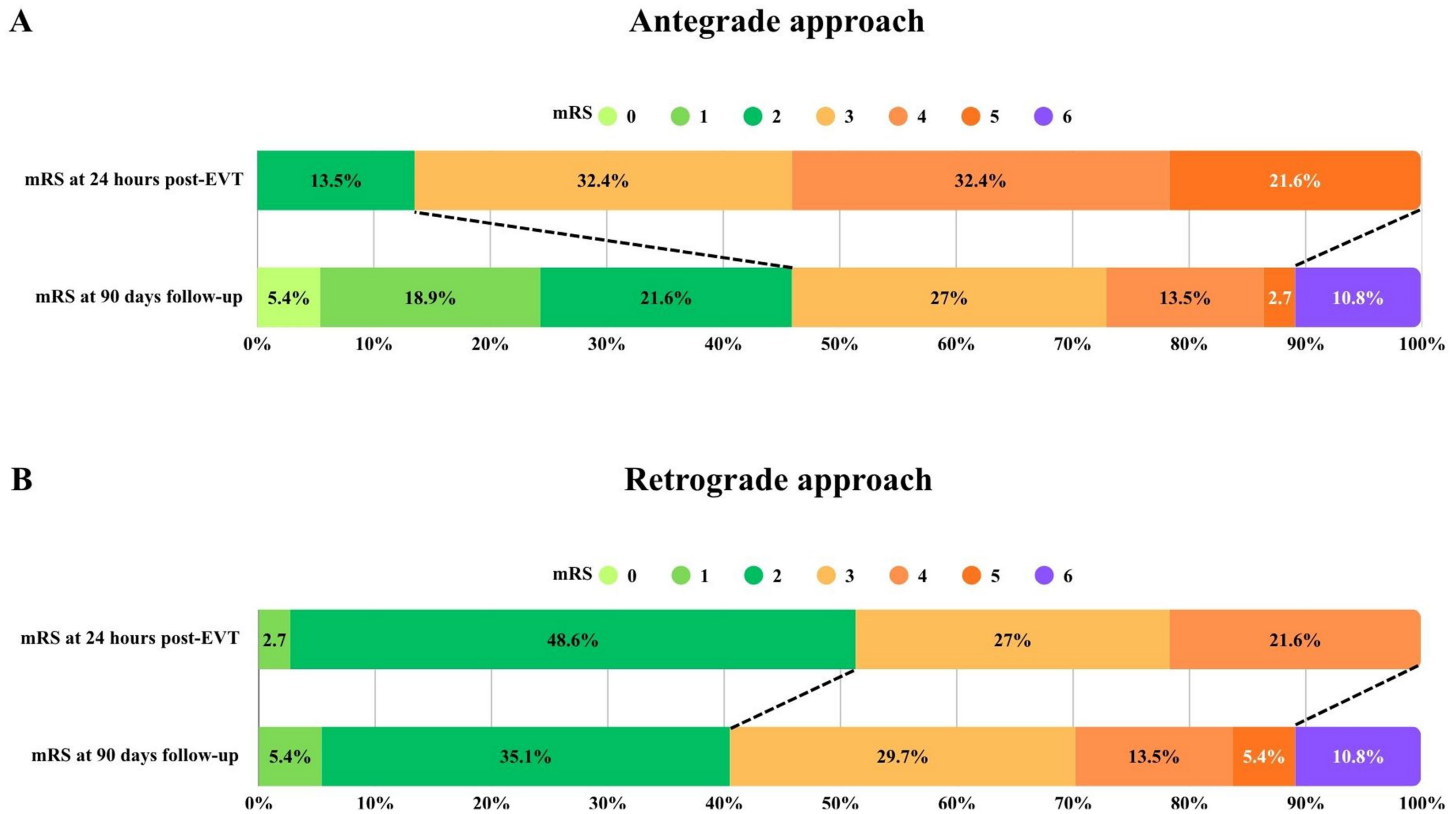
Distribution of modified Rankin Scale (mRS) scores at 24 hours post-procedure and at 90-day follow-up, where the stacked bar charts illustrate the shift in functional outcomes for patients managed with the antegrade approach **(A)** versus the retrograde approach **(B)**, with favorable functional outcome (mRS 0–2) represented by the green segments (EVT, endovascular thrombectomy).

Figure 2 illustrates the proportion of patients achieving a favorable functional outcome, defined as a modified Rankin Scale score of 0 to 2, at 24 h and at 90 days in the two treatment groups. At 24 h after the procedure, 13.5% of patients in the antegrade group reached an mRS score of 0 to 2, compared with 2.7% in the retrograde group. At the 90 day follow up, favorable outcomes increased in both groups, with 45.9% in the antegrade group and 40.5% in the retrograde group.

## Discussion

In our study, the majority of patients were male, a distribution that parallels previous reports of tandem occlusions, including those by Stampfl et al. (87.5%) and Akpinar et al. (80%), although it remained lower than the proportion described by Dang et al. (94.1%) (7, 10, 11). This male predominance is consistent with prior cohorts and may reflect a sex-related trend in tandem lesion epidemiology. The median age of our patients was also lower than that reported by Eker et al. for both antegrade and retrograde strategies, indicating potential demographic differences between populations (12). The rate of patients with hypertension and diabetes was lower than that reported by Eker et al. and Sallustio et al., but higher than that observed in the general Vietnamese population (12–15). This may be explained by the lower prevalence of these conditions in the Vietnamese population compared with American and Italian populations (16, 17). Our study additionally provides the description of intracranial occlusion site distribution in Vietnamese patients with tandem lesions, based on a larger patient cohort than that reported in the study by Luu et al. (7). When compared with the findings of Sallustio et al., our cohort exhibited a slightly higher proportion of M1 occlusions (85.1% vs. 84.7%), a similar proportion of M2 occlusions (10.8% vs. 11.1%), and a higher frequency of combined M1 and M2 involvement (4.1% vs. 0%) (15). Baseline stroke severity, as measured by the NIHSS score, was lower than that reported by Eker et al. but consistent with the values described by Spiotta et al. and Duwicquet et al. (12, 18, 19).

Regarding the immediate outcomes after intervention, our study found that the antegrade approach achieved a slightly higher rate of successful reperfusion than the retrograde approach. Although both strategies demonstrated high overall recanalization rates, this pattern contrasts with the findings of a meta-analysis of 11 studies, in which the retrograde approach was associated with higher reperfusion success (5). Similar advantages of the retrograde technique were also reported by Haussen et al. (6). However, the broader literature remains inconclusive. Several observational studies, including large multicenter analyses from the TITAN investigators and other international groups, have compared intracranial-first (“head-first” or retrograde) and extracranial-first (“neck-first” or antegrade) strategies in tandem occlusions, but the currently available evidence remains insufficient to establish the superiority of either approach (6, 9). Earlier meta-analyses, including that of Wilson et al., likewise reported no statistically significant differences between extracranial-first and intracranial-first approaches, or between extracranial stenting and angioplasty alone (9). Taken together, these findings suggest that immediate procedural outcomes may vary across cohorts and centers, and that the apparent advantage of one strategy may depend on lesion characteristics, technical factors, and peri-procedural management.

In our cohort, residual stenosis after the procedure and the rate of early stent occlusion within 24 h were comparable between groups, suggesting that reconstruction of the cervical carotid lesion was technically achievable with either treatment sequence. This is consistent with prior reports showing that antegrade and retrograde strategies may yield similar technical and angiographic results in selected patients undergoing emergent carotid stenting for tandem lesions (20). However, early neurological improvement at 24 h, reflected by a greater reduction in NIHSS score, appeared more pronounced in the retrograde group.

Comparison with prior observational studies suggests that the conflicting findings in the tandem-lesion literature are likely attributable to substantial methodological heterogeneity. Earlier studies varied in patient selection, imaging criteria, lesion etiology, and procedural workflow, which may partly explain the inconsistent results. In the study by Dong Yang et al., patients with acute tandem occlusion stroke treated with the retrograde approach achieved better functional outcomes than those managed with the anterograde strategy; however, the authors emphasized that randomized trials are still needed to determine the optimal treatment approach (21). These discrepancies may also reflect differences in the proportion of anterior versus mixed-circulation lesions, the inclusion of atherosclerotic disease versus carotid dissection, and variations in acute carotid stenting practice, balloon angioplasty strategy, stent selection, and peri-procedural antithrombotic therapy [Di Donna et al. (1)].

Our findings contribute to the expanding evidence that both antegrade and retrograde strategies are viable options in the treatment of tandem occlusions, but their relative benefits may differ depending on procedural workflows, operator experience, and population characteristics. In this regard, our study adds real-world data from Vietnam to the existing literature, a setting that remains underrepresented in studies of tandem lesion thrombectomy. These results support the notion that no single approach is universally superior, and treatment strategy should be tailored to anatomical considerations and institutional practice patterns.

Nevertheless, earlier pooled evidence has not consistently shown a statistically significant difference in 90-day outcomes between the two treatment sequences. Wilson et al. reported no significant difference in clinical outcome between extracranial-first and intracranial-first approaches, further supporting the interpretation that the comparative effectiveness of the two strategies remains uncertain (9). Our results are therefore not isolated, but rather contribute to an ongoing body of literature in which the direction and magnitude of benefit have varied across studies.

Regarding 90-day clinical outcomes, Table 3 shows that the antegrade and retrograde strategies were associated with similar rates of favorable functional outcome. The two groups also had identical rates of symptomatic intracerebral hemorrhage, while the incidence of delayed stent occlusion was low and comparable between them. The antegrade approach in our study demonstrated a higher proportion of favorable functional outcomes compared with the retrograde strategy, whereas the rates of symptomatic intracerebral hemorrhage and mortality were similar between the two groups. These findings differ from those of Yang et al. (21) and also contrast with results from several other studies. In a prospective cohort of 289 patients with tandem occlusions, the antegrade strategy was associated with lower rates of favorable outcomes, lower sICH rates, and higher mortality compared with the retrograde approach (6). Furthermore, a systematic review and meta-analysis reported that the antegrade technique yielded lower rates of favorable outcomes, higher rates of sICH, and lower mortality than the retrograde technique (5). Collectively, these inconsistencies highlight the considerable heterogeneity across studies evaluating the long-term outcomes of the two procedural strategies.

Regarding the 90-day clinical outcomes, Table 3 shows that the antegrade and retrograde strategies achieved comparable rates of favorable functional recovery, with identical rates of symptomatic intracerebral hemorrhage and similar mortality. Although a slightly higher proportion of patients in the antegrade group attained mRS 0–2 at 90 days, the difference was not statistically significant and does not indicate a clear benefit of either approach.

Further insight into the recovery trajectory is illustrated in Figure 2, which depicts the temporal distribution of functional outcomes. At 24 h post-procedure, early functional independence (mRS 0–2) remained uncommon in both groups—13.5% in the antegrade group and 2.7% in the retrograde group. These early differences likely reflect variations in baseline stroke severity, collateral circulation, or immediate postprocedural physiology, as early mRS values are sensitive to acute clinical fluctuations. By 90 days, both groups demonstrated substantial neurological improvement, with functional independence rising to 45.9 and 40.5% in the antegrade and retrograde groups, respectively. The convergence of these outcomes over time supports the conclusion that long-term recovery is similar regardless of the procedural sequence.

These findings contrast with results from several major studies. Yang et al. and a large multicenter cohort of 289 patients reported that the retrograde strategy was associated with higher rates of functional independence and lower mortality [Yang et al. (6)]. A systematic review and meta-analysis further supported the retrograde approach, identifying higher reperfusion success and superior functional outcomes compared with the antegrade technique (5). However, the persistence of conflicting findings across observational studies has led to the development of prospective randomized trials. TITAN was designed as the first randomized trial to evaluate emergent carotid stenting during thrombectomy in anterior circulation tandem occlusions (6). In addition, EASI-TOC is an ongoing phase III multicenter randomized PROBE trial, and PICASSO is a pragmatic randomized trial evaluating acute carotid stenting strategies in this setting [(22); Kasab et al. (23)]. These studies are expected to provide higher-level evidence regarding cervical carotid management during thrombectomy, although they primarily address the role and timing of acute carotid stenting rather than directly randomizing antegrade versus retrograde sequencing. The divergence between our findings and the existing literature underscores the considerable heterogeneity across studies, including differences in lesion characteristics, operator preference, procedural workflows, peri-procedural antithrombotic strategies, and institutional experience.

Our study has several limitations. First, its retrospective design prevents random allocation of treatment strategy and may introduce selection bias. Second, heterogeneity in endovascular techniques, particularly among patients with atherosclerotic tandem lesions, may affect procedural consistency and limit the comparability of outcomes between the antegrade and retrograde groups. Third, the sample size and number of clinical events were insufficient to support multivariable adjustment or propensity score analysis. Fourth, this was a small single-center study, and therefore its external validity is more limited than that of large multicenter registries and ongoing randomized trials. Accordingly, our findings should be interpreted as complementary real-world data rather than definitive evidence regarding the optimal procedural sequence.

## Conclusion

In our cohort, antegrade and retrograde treatment strategies yielded comparable angiographic and clinical outcomes, including successful reperfusion, early neurological improvement, and 90-day functional recovery. These findings support the feasibility of both approaches in current endovascular practice and add real-world data to the existing literature. However, given the retrospective single-center design and limited sample size, further multicenter randomized controlled trials are needed to determine the optimal procedural sequence for tandem occlusions.

## Data Availability

The raw data supporting the conclusions of this article will be made available by the authors to any qualified researcher upon reasonable request.
