# Supplementary material for: Treatment outcomes of antegrade versus retrograde approaches in tandem occlusion: a single-center retrospective study from Vietnam
Source: Front Neurol. 2026 Jun 24;17:1756539. doi: 10.3389/fneur.2026.1756539 (PMC13341480; doi:10.3389/fneur.2026.1756539)
Supplement: Supplementary file 1 [file Supplementary_file_1.DOCX]

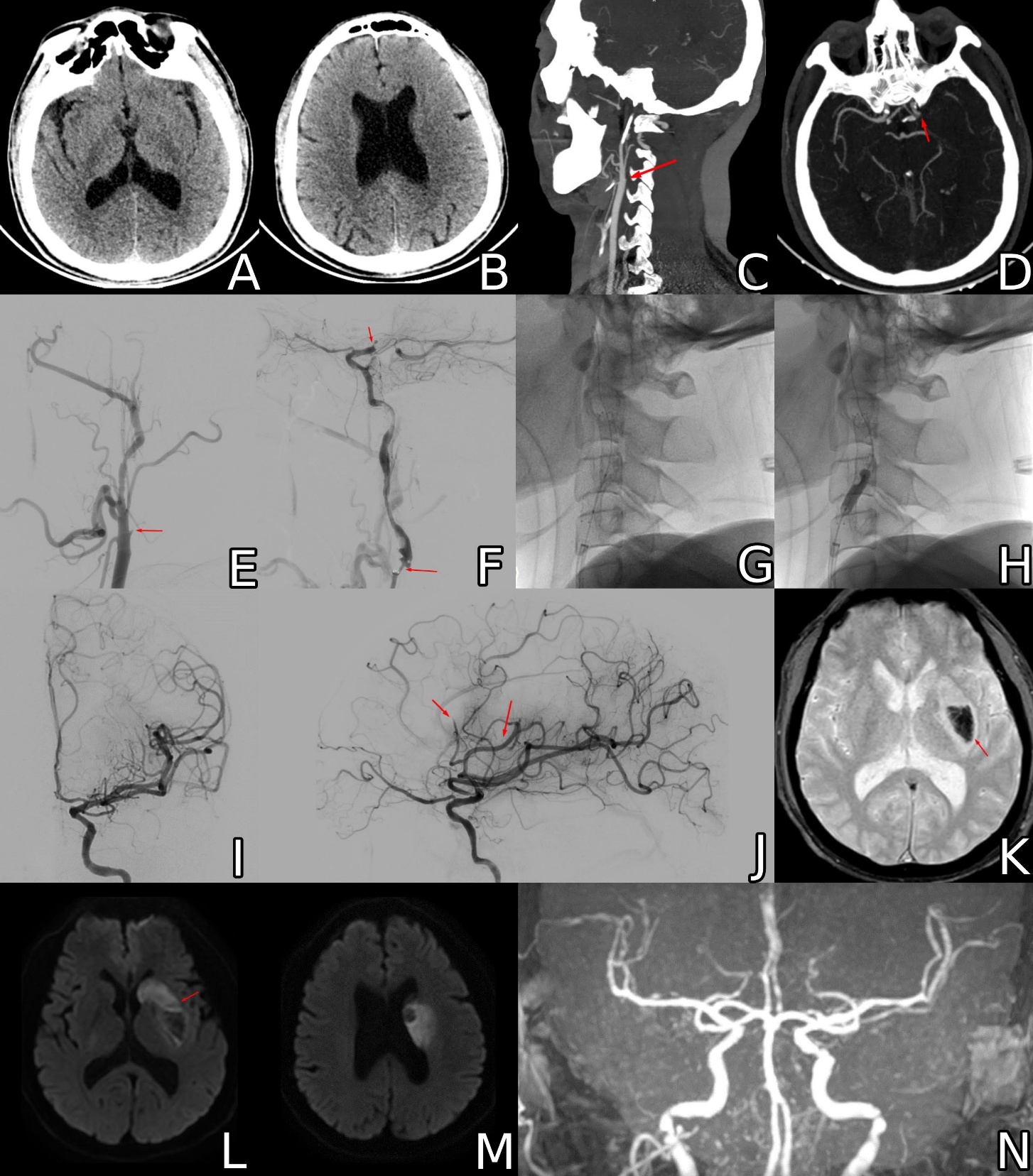


**Case 1:** A 53-year-old male patient was admitted due to confusion and right-sided weakness, with a symptom onset of 3 hours 5 minutes. His NIHSS score was 18 and Glasgow Coma Scale was 12. The patient received intravenous thrombolysis at a dose of 0.9 mg/kg. Non-contrast CT showed an ASPECTS score of 8 (Figure A, B). Multislice CT angiography demonstrated complete occlusion of the left internal carotid artery and left middle cerebral artery (Figure C, D). Figure E shows complete occlusion of the left internal carotid artery on DSA. The device could not be advanced through the stenotic segment, so balloon angioplasty and stent placement were performed at the carotid origin (Figure F, G, H). Subsequently, intracranial thrombectomy was performed; however, clot migration to an M3 branch occurred, with a final reperfusion of TICI 2b (Figure I, J). MRI performed 24 hours later showed petechial hemorrhagic transformation in the putamen (Figure K) and infarction of the deep gray nuclei (Figure L, M). Intracranial arteries were patent on 3D TOF MRA (Figure N). The patient’s motor function improved, and at 90 days his modified Rankin Scale score was 2.


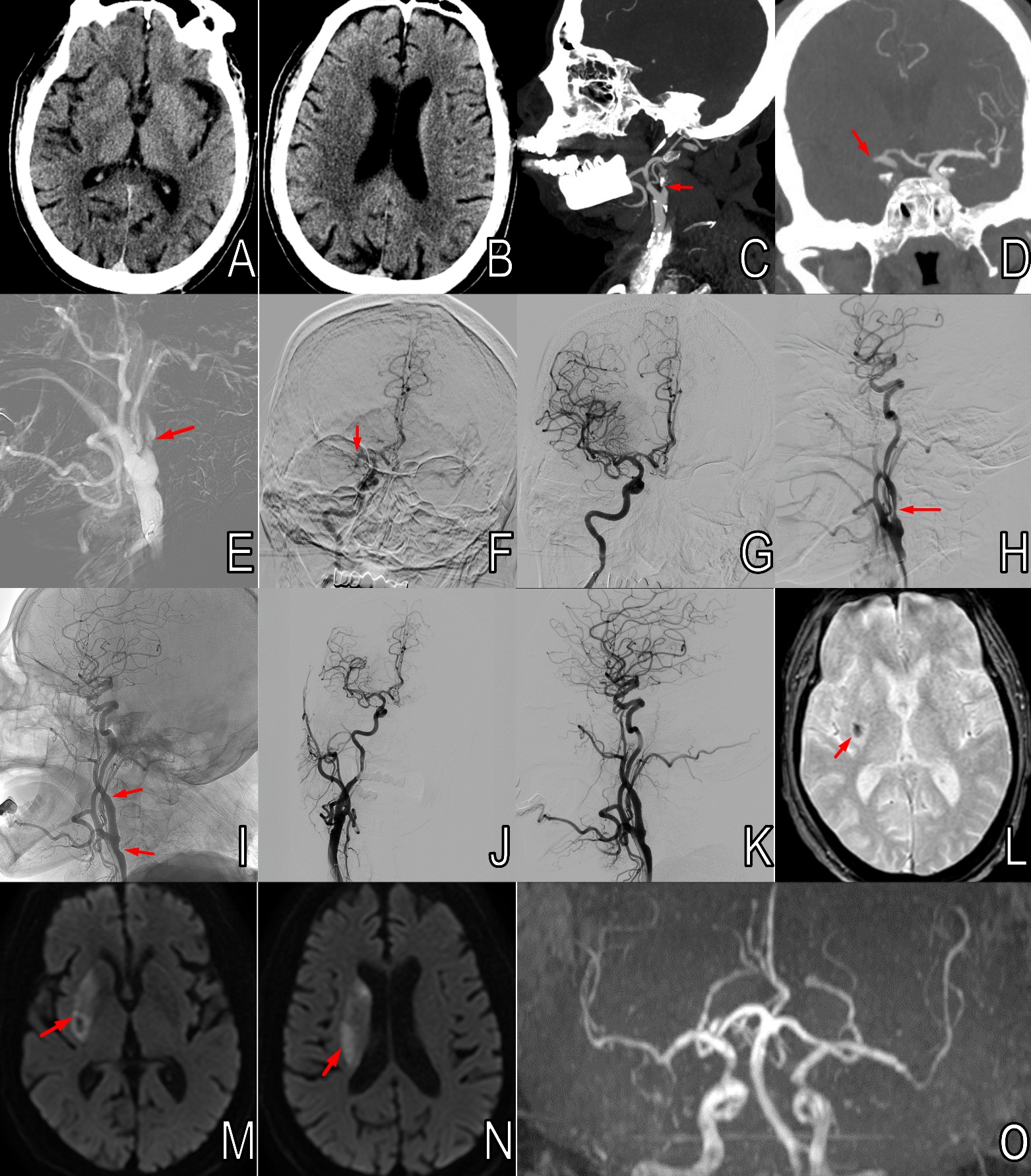


**Case 2:** A 74-year-old male patient was admitted with left-sided hemiplegia, with a symptom onset of 4.5 hours. His NIHSS score was 10 and Glasgow Coma Scale was 14. He presented with left hemiplegia with muscle strength of 1/5. Non-contrast CT (Figure A, B) showed an ASPECTS score of 8. Figures C and D demonstrate right internal carotid artery (ICA) stenosis/occlusion and right middle cerebral artery (MCA) occlusion. DSA confirmed severe stenosis at the right ICA origin (Figure E). After crossing the stenotic segment of the ICA, angiography revealed M1 occlusion of the ICA–MCA segment (Figure F). Mechanical thrombectomy was performed using aspiration alone, achieving complete reperfusion (Figure G). A Porter catheter was then pulled back to the common carotid artery. Follow-up angiography of the carotid origin (Figure H) showed severe (70–80%) ICA origin stenosis. A Protége 6–8/40 stent was deployed across the stenosis, with good stent expansion (Figure I). Post-stenting angiography demonstrated good flow through the stent into the intracranial circulation, with final TICI 3 reperfusion (Figure J, K). MRI performed 24 hours later showed stable infarct lesions (Figure M, N) and a small hemorrhagic transformation in the putamen (Figure L). Intracranial arteries were fully patent on 3D TOF MRA (Figure O). The patient’s muscle strength improved to 3/5. At 90 days, his modified Rankin Scale score was 1.
